# Estrogen and Progesterone Receptor Immunoexpression in Fallopian Tubes among Postmenopausal Women Based on Time since the Last Menstrual Period

**DOI:** 10.3390/ijerph18179195

**Published:** 2021-08-31

**Authors:** Agnieszka Brodowska, Marta Grabowska, Katarzyna Bittel, Sylwester Ciećwież, Jacek Brodowski, Małgorzata Szczuko, Iwona Szydłowska, Małgorzata Piasecka

**Affiliations:** 1 Department of Gynecology, Endocrinology and Gynecological Oncology, Pomeranian Medical University, Unii Lubelskiej 1, 71-256 Szczecin, Poland; agabrod@wp.pl (A.B.); kbittel@o2.pl (K.B.); sylwester.ciecwiez@pum.edu.pl (S.C.); iwona.szydlowska@pum.edu.pl (I.S.); 2 Department of Histology and Developmental Biology, Pomeranian Medical University, Żołnierska 48, 71-210 Szczecin, Poland; martag@pum.edu.pl; 3 Department of Primary Healthcare, Pomeranian Medical University, Żołnierska 48, 71-210 Szczecin, Poland; jacek.brodowski@pum.edu.pl; 4 Department of Human Nutrition and Metabolomics, Pomeranian Medical University in Szczecin, Broniewskiego 24, 71-460 Szczecin, Poland; malgorzata.szczuko@pum.edu.pl

**Keywords:** fallopian tube, estrogen receptor, progesterone receptor, menopause

## Abstract

Existing data on the expression of estrogen receptor (ERα) and progesterone receptor (PR) in fallopian tubes in postmenopausal women are mostly inconclusive. Therefore, we assessed ERα and PR immunoexpression in the oviducts of these women. One hundred postmenopausal women were divided into three groups based on time elapsed since the last menstrual period: (A) 1–5 years, (B) 6–10 years, and (C) ≥11 years. In all groups, both in the glandular epithelium and stroma of the ampulla and isthmus of the oviduct, immunolocalization of ERα and PR were noted. The glandular epithelium of the ampulla showed a higher percentage of PR-positive cells than the isthmus in each group. Regarding ERα, there were no significant differences. In the glandular epithelium in both the ampulla and isthmus, the percentage of ERα- and PR-positive cells was significantly higher than that in the stroma in each study group and higher in the A group than in the C group. In conclusion, in postmenopausal women, time elapsed since the last menstrual period in the fallopian tubes was positively correlated with the following: (1) the epithelium showed vacuolation of cytoplasm with greater frequency, (2) the proportion of ciliated cells decreased, and (3) the percentage of ERα- and PR-positive cells also decreased. The obtained results indicate a significant decrease in ERα and PR expression depending on the time that has elapsed since the last menstruation, which is undoubtedly related to the loss of the reproductive function of the patients.

## 1. Introduction

In recent years, an increase in average length of life has been observed in women, most of which falls in the postmenopausal period. Demographic data have revealed that 25 million women undergo menopause worldwide each year. It is estimated that by 2030, the number of postmenopausal women worldwide could reach 1.2 billion [1]. Therefore, this situation necessitates a detailed analysis of the characteristic issues during this period. As defined, menopause is the last menstrual period followed by no menstruation for the next 12 months, with no pathological causes found. This is a natural process that constitutes one of the signs of aging and is associated with numerous changes in the female body. Menopause is directly related to the end of generative ovarian function, translating into loss of fertility and somatic and psychological changes. Typical symptoms of menopause include vasomotor symptoms (hot flushes, night sweats), vaginal dryness, and sleep disturbances [2,3].

One of the consequences of aging in the female body is a significant decrease in the concentration of sex steroid hormones in the blood due to the gradual decline of ovarian function. Therefore, in the postmenopausal period, total estrogens, including estradiol (E2) and estrone (E1), and progesterone production are significantly reduced. In the postmenopausal period, ovarian hormonal activity is minimal, the concentration of E2 decreases, and estrogens are mainly produced in peripheral tissues (including adipose tissue) as a result of extraglandular aromatization of adrenal androstenedione, which is converted into E1 [4,5].

Estrogens and progesterone play a key role in the development and proper functioning of the female reproductive system, respectively. Of note, these hormones mediate cyclic variation of the fallopian tube epithelium across the ovarian cycle [6]. Estrogens also have a significant effect on the function of other organs, including the cardiovascular and musculoskeletal systems, bone integrity, and behavior [2,7]. These hormones exert numerous beneficial effects on the lipid profile. Estrogens decrease total cholesterol, low-density lipoprotein (LDL), and apolipoprotein A while increasing high-density lipoprotein (HDL) and triglycerides [7]. Moreover, estrogens exert neuroprotective effects by decreasing the risk of stroke, Parkinson’s disease, and Alzheimer’s disease [7]. In postmenopausal women, the protective effect of estrogens (described above) decreases. Progesterone plays a key role mainly in the induction of ovulation, preparation of the endometrium for the implantation process, proper development of a fertilized ovum, and maintenance of pregnancy at the early stage. In addition, progesterone is involved in the reduction of prostaglandin synthesis, in the development of the mammary gland in preparation for milk secretion, and in stimulating bone formation [8,9].

The activity of steroid hormones is manifested through the appropriate receptors present in the target cells. In humans, the presence of these receptors has been found in all organs of the body, but individual tissues differ in their arrangement. In the female reproductive system, estrogen receptors (ERs) and progesterone receptors (PRs) are found in the vagina, uterus, fallopian tubes, and ovaries [4,10,11].

Estrogen receptors belong to the nuclear receptors family. They have two major subtypes, ERα and ERβ, which differ functionally and structurally, and their tissue location is distinct. Both human subtypes are encoded by genes located on different chromosomes. The ERα protein has a molecular size of 66 kDa, while the ERβ protein has a molecular size of 54 kDa [12]. Both proteins consist of six domains which are functionally distinct. The characteristic composition of amino acids in the individual structural regions gives properties specific to the selected subtype in the transmission of E2 signaling. Depending on the balance between ERα and ERβ activities in target cells, estrogen signaling is appropriately stimulated or inhibited [10]. It is worth mentioning that the tissue distribution of both receptor subtypes shows species-specific differences. However, ERα has been suggested to be essential in mediating E2 signaling in the uterus, ovarian theca cells, pituitary gland, mammary glands, testes, epididymis, prostate stroma, skeletal muscle, and bone and adipose tissue. In turn, ERβ is essential in the ovarian granulosa cells, bladder, colon, lungs, and epithelium of the prostate. Additionally, both subtypes were found in the cardiovascular and central nervous systems [10,11,12]. It should be highlighted that in individual tissues, the expression of each subtype is specific to a given cell type. In the uterus and fallopian tube, ERα activity is cyclically regulated by hormone levels [13]. In rodents, ERα is a key regulator in fallopian tube development. Alternatively, ERβ is likely involved in the regulation of the calcium-dependent ciliated beating of the fallopian tube mediated by estrogen. However, the significance of ERα in the action of estrogens related to the production and secretion of proteins in the fallopian tubes has not yet been established [14,15,16].

There are available reports on the immunolocalization and immunoexpression of ER and PR in the fallopian tubes of premenopausal women [17,18,19,20,21,22,23,24]. However, according to current knowledge, there are only a few reports on the expression of ERα and PR in normal human fallopian tubes after menopause [17,18,19,20]. Some authors have revealed that in the fallopian tube of postmenopausal women, ERα and PR expression decreased or remained virtually undetectable, while other researchers have found that ERα and PR expression increased [17,18,20]. In turn, other studies showed weak expression of ERα and strong expression of PR [19]. Unfortunately, obtained data are inconclusive. Therefore, we decided to assess the morphology, immunolocalization, and immunoexpression of ERα and PR in the fallopian tubes of postmenopausal women and compare them in different segments of the fallopian tube, depending on the time elapsed since the last menstrual period.

## 2. Materials and Methods

### 2.1. Patients

The study included 100 female patients who underwent surgery due to benign uterine and/or ovarian neoplasms at the Department of Gynecology and Urogynecology, Pomeranian Medical University in Szczecin in 2012–2013. Only the patients in whom at least one year elapsed since the last menstrual period qualified for the study. The exclusion criteria of the study were as follows: (1) iatrogenic amenorrhea, (2) retained ovarian function, (3) use of menopausal hormone therapy, and (4) history of endocrine disorders, malignancies, or surgeries that might impair perfusion of the adnexa (ovaries and fallopian tubes).

Study participants were divided into three groups (A, B and C) based on the time elapsed between the last menstrual period (LMP) and surgery: 1–5 years for group A (*n* = 40), 6–10 years for group B (*n* = 30), and ≥11 years for group C (*n* = 30). Prior to the beginning of the research, the authors received the approval of the Ethics Committee of Pomeranian Medical University in Szczecin.

### 2.2. Histological Analysis

All patients underwent total or subtotal hysterectomy with removal of the adnexa or solely fallopian tubes. The obtained fallopian tubes were routinely fixed in 4% buffered paraformaldehyde and then embedded in paraffin blocks for further analysis. Subsequently, using a microtome, 3 µm-thin sections were cut and placed on polylysine-coated slides. After deparaffinization and rehydration, fallopian tube specimens were stained using standard methods. Hematoxylin and eosin (H&E) staining was performed according to a protocol described in detail by Bancroft and Gamble (2002) [25].

### 2.3. Immunohistochemistry

Immunostaining of paraffin-embedded fallopian tubes was performed following the manufacturer’s guidelines (Dako, Glostrup, Denmark). The sections of the fallopian tubes were deparaffinized and rehydrated. Antigen retrieval was performed by boiling the slides for 30 min in Target Retrieval Solution Citrate (Dako, Glostrup, Denmark) at pH 6.0 (for PR) and in Target Retrieval Solution (Dako, Glostrup, Denmark) at pH 9.0 (for ERα). Endogenous peroxidase was blocked using Peroxidase-Blocking Solution (Dako, Glostrup, Denmark) for 10 min at room temperature. To determine the immunoexpression of ERα and PR, the following primary antibodies were used: (1) rabbit monoclonal antibody IgG against estrogen receptor α (clone: EP1; Dako, Glostrup, Denmark), diluted 1:100; (2) mouse monoclonal antibody IgG against progesterone receptor (clone: PgR 636, which reacts with the PR-A and PR-B forms; Dako, Glostrup, Denmark), diluted 1:100. The slides were incubated with the primary antibodies in a humid chamber at room temperature for 30 min. Subsequently, the slides were incubated with a complex containing a secondary antibody conjugated with horseradish peroxidase (Dako, Glostrup, Denmark). Next, diaminobenzidine was applied. At the final step, the specimens were washed in distilled water, counterstained with Mayer’s hematoxylin (Sigma-Aldrich Co., St Louis, MO, USA), dehydrated, and coverslipped. After each stage, the slides were washed in phosphate-buffered saline (PBS). Negative controls for reaction specificity were also performed. The obtained specimens were examined under a light microscope (Olympus BX 41, Hamburg, Germany).

### 2.4. Quantitative Analysis of Immunoexpression of ERα and PR

ERα- and PR-immunostained slides were scanned at a magnification of 400× (resolution of 0.25 μm/pixel) using the ScanScope AT2 scanner (Leica Microsystems, Wetzlar, Germany). The obtained digital images of the slides were analyzed using the ImageScope viewer (Version 11.2.0.780; Aperio Technologies, Vista, CA, USA). Immunoexpression of ERα and PR in the glandular epithelium and the stroma of the ampulla and isthmus of the fallopian tubes was expressed as a percentage of ERα- and PR-immunopositive cells using automatic computer analysis. For this assessment, a nuclear v9 algorithm (version 9.1; Aperio Technologies, Vista, CA, USA) was applied. Analyzed areas were manually determined. Using the algorithm, the percentage of cells with ERα-positive and PR-positive immunostaining was independently counted in 40 random fields in each group with an average area of 0.4 mm^2^.

### 2.5. Statistical Analysis

The results were analyzed using Statistica 13.1 software (StatSoft, Kraków, Poland). The arithmetical means, SDs (X ± SD), medians, and minimum and maximum values were calculated. The quantitative values were first analyzed for normality using the Shapiro–Wilk test. The results of calendar age, age at the last menstrual period, and body mass index revealed a normal distribution, and parametric analysis of variance (ANOVA) was used. For ERα and PR expression results, as most of the distributions deviated from a normal distribution, to assess the differences between the groups, a nonparametric Kruskal–Wallis test with Dunn’s multiple comparison test for post hoc analysis was used. Intergroup differences were considered significant at *p* < 0.05.

## 3. Results

### 3.1. Baseline Characteristics of the Patients

The three groups of patients were statistically significantly different regarding their calendar ages (*p* < 0.001) and body mass index (BMI) (*p* < 0.001). The patients in the C group were the oldest and had the highest BMI. There was no statistical significance in the age at last menstrual period between the compared groups (Table 1).

### 3.2. Morphological Studies

**Figure 1:**
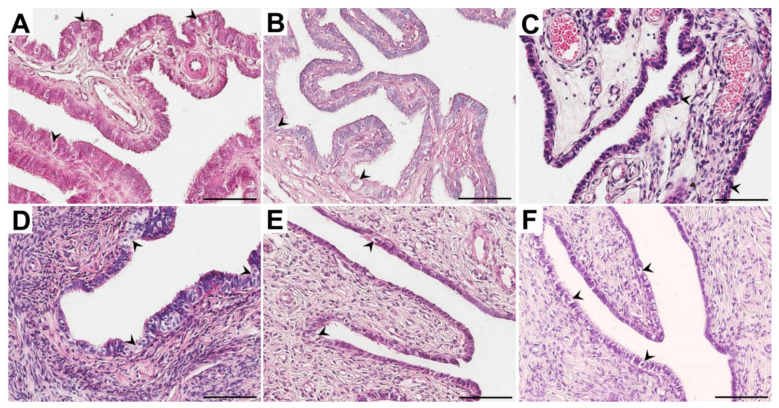
Representative light micrographs of the hematoxylin and eosin-stained ampulla (**A**–**C**) and isthmus (**D**–**F**) of the fallopian tubes in postmenopausal women for whom 1–5 years (**A**,**D**), 6–10 years (**B**,**E**) and ≥ 11 years (**C**,**F**) had elapsed between the last menstrual period and surgery. Note that in the glandular epithelium, vacuolization was observed (black arrowheads). Scale bar—50 µm.

In all groups of women, the fallopian tubes were mostly lined with a single columnar epithelium. In the tubal epithelial cells, oval or elongated nuclei were observed. Epithelial cells were often characterized by different heights, and in some areas, the epithelium appeared pseudostratified. In the ampulla, the tubal epithelial cells were predominantly columnar, while in the isthmus, they were rather varied. In all groups of women, some cells were characterized by numerous cilia at the luminal surface (ciliated cells), while nonciliated cells were also recognized. In the isthmus, fewer ciliated cells than in the ampulla were observed. Moreover, in both the ampulla and the isthmus, the proportion of ciliated cells decreased with time since the last menstrual period. In all groups of women, in some areas, the epithelium showed cytoplasm vacuolation, with the highest frequency in the C group (Figure 1).

### 3.3. Immunolocalization and Immunoexpression of ERα

**Figure 2:**
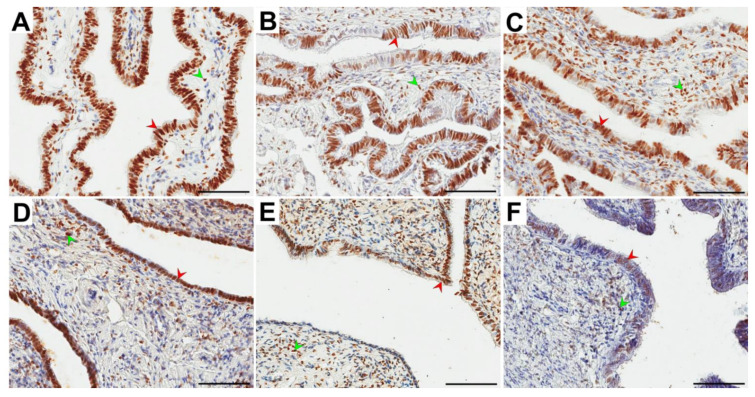
Representative light micrographs of the nuclear immunolocalization (brown color) of estrogen receptor α (ERα) in the glandular epithelium (red arrowheads) and the stroma (green arrowheads) of the ampulla (**A**–**C**) and isthmus (**D**–**F**) of the fallopian tubes in postmenopausal women for whom 1–5 years (**A**,**D**), 6–10 years (**B**,**E**) and ≥ 11 years (**C**,**F**) had elapsed between the last menstrual period and surgery. Note that in the glandular epithelium, immunonegative ERα cells in the form of clusters were usually observed. Scale bar—50 µm.

In all groups of women, ERα immunolocalization in the ampulla and isthmus of the fallopian tube in the form of brown-stained cell nuclei in both the glandular epithelium and stroma was observed. Immunopositive ERα cells usually formed clusters composed of several cells spread among ERα-negative cells in the glandular epithelium (Figure 2).

In the epithelium and stroma, the obtained percentages of ERα-immunopositive cells had a similar distribution in the ampulla and isthmus of the fallopian tubes. In both the glandular epithelium and the stroma, there was no statistically significant difference in the percentage of ERα-positive cells in the ampulla vs. isthmus for each group (Table 2). In both the ampulla and isthmus, the percentage of ERα-positive cells in the glandular epithelium in the A group was statistically significantly different (*p* = 0.013 and *p* = 0.003, respectively) only vs. the C group. The percentage of these cells in the A group was higher than that in the C group (medians: 85.8% vs. 73.4% and 84.2% vs. 76.8%, respectively). There was no statistically significant difference in the percentage of ERα-positive cells in the stroma between all compared groups (Table 2). In the percentage of ERα-positive cells between the stroma and glandular epithelium in each group, a significant difference (*p* < 0.001) was noted. In the stroma, a lower percentage of ERα-positive cells was revealed (Table 2).

### 3.4. Immunolocalization and Immunoexpression of PR

**Figure 3:**
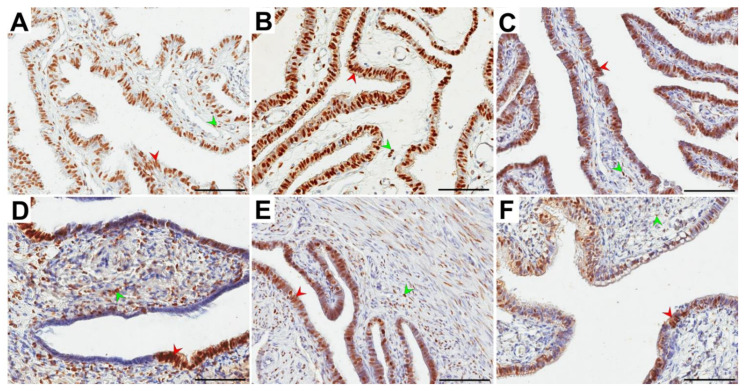
Representative light micrographs of the nuclear immunolocalization (brown color) of progesterone receptor (PR) in the glandular epithelium (red arrowheads) and the stroma (green arrowheads) of the ampulla (**A**–**C**) and isthmus (**D**–**F**) of the fallopian tubes in postmenopausal women for whom 1–5 years (**A**,**D**), 6–10 years (**B**,**E**) and ≥11 years (**C**,**F**) had elapsed between the last menstrual period and surgery. Note that in the glandular epithelium, immunonegative PR cells in the form of clusters were usually observed. Scale bar—50 µm.

In all groups of women, PR immunolocalization in the ampulla and isthmus of the fallopian tube was characterized by brown-stained cell nuclei in both epithelial and stromal cells. Immunopositive and negative PR cells usually formed clusters of over a dozen or more cells in the glandular epithelium (Figure 3).

In the epithelium and stroma, the obtained percentages of PR-immunopositive cells had similar distributions for both the ampulla and isthmus. In the glandular epithelium, the percentage of PR-positive cells was significantly higher (*p* < 0.001) in the ampulla vs. isthmus for each group. In the stroma, there was no statistically significant difference in the percentage of these cells (Table 3).

In both the ampulla and isthmus, the percentage of PR-positive cells in the glandular epithelium in the A group was statistically significantly different (*p* = 0.027 and *p* = 0.003, respectively) only vs. the C group. The percentage of these cells in the A group was higher than that in the C group (medians: 86.4% vs. 74.8% and 75.0% vs. 55.9%, respectively). There was no statistically significant difference in the percentage of PR-positive cells in the stroma between all compared groups (Table 3).

In the percentage of PR-positive cells between the stroma and glandular epithelium in each group, a significant difference (*p* < 0.001) was revealed. In the stroma, a lower percentage of PR-positive cells was noted (Table 3).

## 4. Discussion

This study focused on assessing ERα and PR immunolocalization and immunoexpression in the fallopian tubes and the morphology of tubal epithelial cells in postmenopausal women depending on the time elapsed since the last menstrual period. This is the first report on the characterization of age-related postmenopausal morphological changes and immunoexpression of ERα and PR in normal human fallopian tubes.

We showed that in both the ampulla and the isthmus, the proportion of ciliated cells decreased with time since the last menstrual period (Figure 1). The obtained findings are consistent with the observations of other authors [26,27,28,29,30] and confirmed that the tubal epithelium undergoes sex hormone-dependent changes. While ciliated cells were shown to outnumber secretory cells at the level of the abdominal orifice in women of reproductive age, an inverse proportion of these two cell types was documented in the isthmus. Furthermore, the researchers highlighted that the surface activity of secretory cells was increased inversely to the distance from the uterine orifice. In turn, nonciliated cells were most active during the preovulatory phase of the ovarian cycle, when their cell membranes formed finger- or dome-shaped protrusions on the luminal surface to release their superficial glycoprotein granules in an apocrine manner or via exocytosis [26,27]. Researchers conducting morphological studies confirmed that insufficient hormonal stimulation after menopause results in atrophy of the tubal epithelium, namely, a gradual decrease in the number of ciliated cells and functional impairment of secretory cells [28,29]. It has been found that ciliated cells undergo progressive flattening, and microplicae-like structures can be observed on their surfaces [29,30].

In our studies we have also revealed that in the postmenopausal women the tubal epithelium showed cytoplasm vacuolation in many areas, mainly in postmenopausal women for whom over 11 years elapsed between the last menstrual period and surgery. In women for whom 1–5 and 6–10 years had elapsed since the last menstrual period, cytoplasm vacuolation was noted with the lower frequency (Figure 1). Other authors have noted similar observations [27]. Crow et al. (1994) showed that the tubal epithelium in some sections were characterized by the vacuolation of cytoplasm. The authors stated that vacuolation was associated with accumulation of glycogen particles and occasionally lipid droplets [27]. In our studies the high vacuolation rate may be the result of many age-related degenerative changes (including the accumulation of glycoproteins, lipids, and fluids) and/or alterations associated with cell death. 

In our study, we found immunolocalization of ERα and PR in the glandular epithelium and stroma of the ampulla and isthmus of the oviduct (Figure 2 and Figure 3). This finding is consistent with other reports revealing ER and PR immunolocalization in fallopian tubes of both pre- and postmenopausal women. Expression of ER and PR was demonstrated in the nuclei of ciliated and nonciliated cells of the fallopian tubes, as well as in the tubal stroma and smooth muscle cells [17,18,20,21,22,24,31,32,33,34,35,36,37].

In our study, the glandular epithelium of the ampulla showed a higher percentage of PR-positive cells than the isthmus in each group (Table 3). Alternatively, for ERα, there were no significant differences (Table 2). Urabe et al. (2017) showed that PR expression was significantly decreased in the ampulla of the fallopian tube in postmenopausal women compared to the premenopausal group [20]. We revealed that in the glandular epithelium in both the ampulla and isthmus, the percentage of ERα- and PR-positive cells was significantly higher than that in the stroma in each study group. Moreover, we noted a higher percentage of ERα- and PR-positive cells in postmenopausal women for whom 1–5 years had elapsed between the last menstrual period and surgery versus those for whom over 11 years had elapsed (Table 2 and Table 3). Only a few previous studies analyzed the localization and expression of ERα and PR in human fallopian tubes after menopause [17,18,19,20]. According to the literature, postmenopausal women present with a low density of ERα and PR. However, Amso et al. [17] showed that the expression levels of ERα and PR were slightly higher in the ampulla and infundibulum, respectively. According to Shah et al. [18], the expression of ERα in postmenopausal women is weak, whereas the expression of PR remains virtually undetectable. In turn, Punnonen and Lukola [19] demonstrated that postmenopausal women present with weak tubal expression of ERα and strong expression of PR.

Studies of the immunolocalization and immunoexpression of ERα and PR in the fallopian tubes of premenopausal women showed that the intensity of ER expression, already strong during the follicular phase, increases further during the preovulation phase and then decreases in the luteal phase [17,18,19]. An opposite expression pattern, i.e., mid-cycle increase persisting during the luteal phase, was observed for PR. Moreover, the expression of ER and PR in the fallopian tubes of premenopausal women was segment specific. Whereas ER is expressed predominantly in the isthmus and ampulla, the expression of PR is found mainly in the infundibulum and fimbria [17,18,19]. However, some authors showed that both ER and PR are expressed predominantly in the ampulla [21,22] or did not find significant differences in the intensity of ER expression across various tubal segments [19,23]. Similarly, while some researchers documented only minor fluctuations in the immunoexpression of ER during various phases of the menstrual cycle [19,23], others observed strong expression of both ER and PR during the proliferation phase and a subsequent marked decrease during the secretory phase [13]. Finally, one study analyzed the immunoexpression of ER and PR in the fallopian tubes of women with concomitant ectopic pregnancy. Half of these patients showed expression of ER but not PR [24].

In recent years, there has been a decrease in the frequency of hysterectomy procedures as part of the conservative treatment of benign diseases of female genital organs. During this procedure, one of the most important aspects is the decision to remove or retain other structures related to the uterus, such as the fallopian tubes. Until recently, being over 40 years old was one of the main factors qualifying patients who underwent hysterectomy for removal of the ovaries and fallopian tubes. Currently, according to the latest recommendations, the decision to remove the fallopian tubes and ovaries is made based on the analysis of many factors that may be important for patients in the later stages of life [38,39]. Even after patients reach menopausal age, certain gonadal functions are preserved, and hormone replacement therapy is not always advisable. However, preserving the fallopian tubes during hysterectomy has no known benefit. The hormonal profile does not change after salpingectomy, and blood supply to the ovaries is preserved [40]. Because the plugged remains of the fallopian tubes may be associated with later pathologies of this organ, hysterectomy and bilateral salpingectomy are widely used. Unfortunately, the long-term effects of menopausal time in patients undergoing hysterectomy combined with salpingectomy have not been analyzed. Therefore, the negative effects of this treatment are still under discussion.

One of the arguments in favor of removal of the fallopian tubes is the reduction of the risk of developing serous cancer of the ovary, fallopian tube and peritoneum [41]. Available data suggest that fallopian tubes may be the origin of ovarian cancer and might also play a role in the pathogenesis of ovarian serous carcinoma (SC). Because ovarian cancer has a high mortality rate, currently in women who have completed childbearing age and are at risk of ovarian cancer, removal of the fallopian tubes (bilateral salpingectomy) is recommended [42]. Early cancerous lesions were detected in the tubal fimbria of BRCA mutation carriers subjected to prophylactic adnexectomy. These lesions, referred to as serous tubal intraepithelial carcinoma (STIC), were found in 2–17% of the patients, nearly exclusively in the tubal mucosa. Interestingly, tubal involvement and STIC were also demonstrated in 35–70% of women operated on for ovarian or peritoneal high-grade (HG)-SC. In all these patients, cells from tubal and ovarian/peritoneal lesions harbored TP53 mutations and showed similar mitotic activity [43,44]. One proposed mechanism of ovarian carcinogenesis is the implantation of fimbrial epithelial cells to disrupt the ovarian epithelium at the time of ovulation and their subsequent transformation to HG-SC. Indeed, detailed analysis of the ovarian surface epithelium (OSE) showed that it consists of two distinct types of epithelial cells, and approximately 4% of OSE cells show typical reactivity of the tubal epithelium (calretinin+/PAX8-/tubulin), which also supports the implantation hypothesis. Morphologically, however, these cells resemble normal ovarian epithelium. Additionally, an analysis of inclusion cysts showed that up to 78% of them may derive from tubal epithelium. Similar to ovarian SC cells, tubal epithelial cells are characterized by a markedly higher proliferation index than normal ovarian epithelium. Histological evaluation of consecutive stages of ovarian carcinogenesis, i.e., inclusion cysts, cystadenomas, borderline tumors, and low-grade (LG)-SCs, revealed a decrease in the number of ciliated cells in favor of secretory cells, implying that similar to HG-SC, LG-SC may be a consequence of secretory cell expansion [44]. Of note, a hysterectomy itself also contributes to a significant reduction in the risk of malignant neoplasm development within the appendages. However, removal of the uterus reduces the ovarian reserve or accelerates the extinction of gonadal activity [45,46].

In the reproductive period in women, the main functions of the fallopian tube include participation in the process of gamete fertilization, transport of an early embryo to the uterus, and establishment of a normal intrauterine pregnancy [47]. After menopause, no function of the fallopian tubes has been observed in a woman’s body. Research conducted by us and other authors has shown the presence of ERα and PR in the fallopian tubes of postmenopausal women, demonstrating that the fallopian tube is still steroid-responsive tissue. Through the mentioned receptors, the activity of steroid hormones might still manifest after menopause.

To summarize, the available data on the expression and localization of ERα and PR in human fallopian tubes revealed contradictory results and are mostly inconclusive. Therefore, this problem should be a subject of further extensive research involving larger groups of patients.

## 5. Conclusions

In conclusion, in postmenopausal women, time elapsed since the last menstrual period was associated with the following in the fallopian tubes: (1) the epithelium showed more cytoplasm vacuolation, (2) the proportion of ciliated cells decreased, and (3) the percentage of ERα- and PR-positive cells also decreased. The obtained results indicate a significant decrease in ERα and PR expression depending on the time that has elapsed since the last menstruation, which is undoubtedly related to the loss of the reproductive function of the patients.

## Figures and Tables

**Table 1:** Comparison of calendar age, age at the last menstrual period and body mass index in the study groups.

Parameter	Group (X ± SD)		
	A (*n* = 40)	B (*n* = 30)	C (*n* = 30)
Calendar age (years)	53.6 ^a^ ± 2.4	57.9 ^a^ ± 4.6	67.3 ^a^ ± 7.3
Age at LMP (years)	50.8 ± 4.4	50.2 ± 3.3	50.7 ± 3.9
BMI (kg/m^2^)	24.6 ^b^ ± 3.0	28.5 ^b^ ± 4.6	29.2 ^b^ ± 6.4

A—women for whom 1–5 years had elapsed since the last menstrual period; B—women for whom 6–10 years had elapsed since the last menstrual period; BMI—body mass index; C—women for whom ≥11 years had elapsed since the last menstrual period; LMP—last menstrual period; *n*—number of patients; X ± SD—mean ± standard deviation; common superscripts denote significant differences between compared groups: *p* < 0.001 (^a,b^) (ANOVA).

**Table 2:** The percentage of ERα-positive cells in the glandular epithelium and the stroma of the fallopian tube ampulla and isthmus of postmenopausal women stratified according to time elapsed since the last menstrual period.

Group		Glandular Epithelium Median (Range) X ± SD	Stroma Median (Range) X ± SD
A	ampulla	85.8 ^a,c^ (30.8−95.9) 81.2 ± 12.8	49.8 ^c^ (25.0−75.4) 52.4 ± 13.1
B		81.3 ^d^ (34.8−90.6) 76.6 ± 12.9	50.6 ^d^ (26.1−75.4) 49.5 ± 15.2
C		73.4 ^a,e^ (34.1−89.7) 68.5 ± 13.9	51.5 ^e^ (22.0−71.1) 46.4 ± 14.4
A	isthmus	84.2 ^b,f^ (68.2−94.0) 83.3 ± 6.9	57.0 ^f^ (25.2−72.9) 53.6 ± 13.2
B		83.9 ^g^ (35.1−91.6) 78.6 ± 14.8	56.4 ^g^ (24.8−72.9) 51.7 ± 15.5
C		76.8 ^b,h^ (24.4−92.6) 68.9 ± 18.1	46.4 ^h^ (29.5−71.0) 51.1 ± 13.6

A—women for whom 1–5 years had elapsed since the last menstrual period; B—women for whom 6–10 years had elapsed since the last menstrual period; C—women for whom ≥11 years had elapsed since the last menstrual period; ERα—estrogen receptor alpha; X ± SD—mean ± standard deviation; common superscripts denote significant differences between compared groups: *p* = 0.013 (^a^), *p* = 0.003 (^b^), *p* < 0.001 (^c–h^) (Kruscall–Wallis test).

**Table 3:** The percentage of PR-positive cells in the glandular epithelium and the stroma of the fallopian tube ampulla and isthmus of postmenopausal women stratified according to time elapsed since the last menstrual period.

Group		Glandular Epithelium Median (Range) X ± SD	Stroma Median (Range) X ± SD
A	ampulla	86.4 ^a,c,f^ (68.3−98.8) 85.6 ± 7.5	53.6 ^f^ (37.3−81.4) 54.2 ± 12.2
B		86.0 ^d,g^ (56.2−94.8) 83.7 ± 8.6	52.0 ^g^ (34.0−80.8) 51.8 ± 8.1
C		74.8 ^a,e,h^ (51.7−91.3) 72.2 ± 13.4	48.7 ^h^ (26.4−69.9) 49.4 ± 11.0
A	isthmus	75.0 ^b,c,i^ (51.0−91.8) 73.2 ± 11.7	48.5 ^i^ (25.9−75.8) 50.4 ± 11.4
B		68.3 ^d,j^ (47.7−80.2) 65.7 ± 10.2	47.8 ^j^ (21.7−64.0) 47.2 ± 11.4
C		55.9 ^b,e,k^ (41.1−80.1) 59.6 ± 10.1	45.0 ^k^ (21.5−65.4) 45.7 ± 11.2

A—women for whom 1–5 years had elapsed since the last menstrual period; B—women for whom 6–10 years had elapsed since the last menstrual period; C—women for whom ≥11 years had elapsed since the last menstrual period; PR—progesterone receptor; X ± SD—mean ± standard deviation; common superscripts denote significant differences between compared groups: *p* = 0.027 (^a^), *p* = 0.003 (^b^), *p* < 0.001 (^c–k^) (Kruscall–Wallis test).

## Data Availability

The data presented in this study are available on the reasonable request from the corresponding author.
